# Cardiac Device Therapy in Patients with Chronic Kidney Disease: An Update

**DOI:** 10.3390/jcm13020516

**Published:** 2024-01-17

**Authors:** Bogdan Caba, Laura Vasiliu, Maria Alexandra Covic, Radu Sascau, Cristian Statescu, Adrian Covic

**Affiliations:** 1 Faculty of Medicine, “Grigore T. Popa” University of Medicine, 700115 Iasi, Romania; bogdancaba65@gmail.com (B.C.); laura.tapoi@yahoo.com (L.V.); radu.sascau@gmail.com (R.S.); cstatescu@gmail.com (C.S.); accovic@gmail.com (A.C.); 2 Cardiology Department, Cardiovascular Diseases Institute “Prof. Dr. George I. M. Georgescu”, 700503 Iasi, Romania; 3 Nephrology Department, Dialysis and Renal Transplant Center, “Dr. C.I. Parhon” University Hospital, 700503 Iasi, Romania

**Keywords:** CKD, HF, CRT, LVAD, ICD, CIED, CRT-D

## Abstract

Cardiovascular diseases (CVDs) and chronic kidney disease (CKD) are frequently interconnected and their association leads to an exponential increase in the risk of both fatal and non-fatal events. In addition, the burden of arrhythmias in CKD patients is increased. On the other hand, the presence of CKD is an important factor that influences the decision to pursue cardiac device therapy. Data on CKD patients with device therapy are scarce and mostly derives from observational studies and case reports. Cardiac resynchronization therapy (CRT) is associated with decreased mortality, reduced heart failure symptoms, and improved renal function in early stages of CKD. Implantable cardioverter defibrillators (ICDs) are associated with a significant reduction in the mortality of CKD patients only for the secondary prevention of sudden cardiac death. Cardiac resynchronization therapy with defibrillator (CRT-D) is preferred in patients who meet the established criteria. The need for cardiac pacing is increased three-fold in dialysis patients. CKD is an independent risk factor for infections associated with cardiac devices.

## 1. Introduction

Cardiovascular diseases (CVDs), particularly heart failure (HF), are the leading cause of morbidity and mortality in patients with chronic kidney disease (CKD) [1]. Moreover, the relationship between these two conditions is bidirectional, as the evolution of one condition influences the development of the other and vice versa [1,2]. The progression of CKD is one of the main determinants of a worse prognosis in cardiac disorders, as decreased renal function is associated with increased hospital admissions and deaths [3]. CKD also promotes the occurrence of arrhythmias, sudden cardiac death through electrolyte disorders, left ventricular hypertrophy, myocardial fibrosis, uremic status, or sympathetic overactivity [4]. CKD patients have an increased prevalence of atherosclerosis, vascular calcifications, and vascular stiffness, all which contribute to increased cardiovascular morbidity and mortality. The accumulation of uremic toxins in the advanced stages of CKD promote systemic inflammation and oxidative stress, leading to endothelial dysfunction [5].

Cardiac implantable electronic device (CIED) therapy includes pacemakers, implantable cardioverter defibrillators (ICD), and cardiac resynchronization therapy (CRT). The current indications for each therapy are detailed in the designated subchapters below. The use of CIEDs has increased in the past years [6]. Severe renal impairment is an important element to be considered when choosing the type of CIED/cardiac assist device treatment in patients with HF or who are in need of cardiopulmonary support. The safety and efficacy of these therapies in CKD patients is still questionable due to the lack of evidence caused by their exclusion from large clinical trials [1,2]. Also, end-stage renal disease (ESRD) and dialysis are risk factors for systemic CIED infections due to the constant need for vascular access or altered immune response [7]. On the other hand, dyssynchrony caused by right ventricular pacing can cause pacing-induced HF with reduced left ventricular systolic function, which leads to low cardiac output, inadequate renal perfusion, and decreased renal function [8,9].

The decision to implant cardiac devices is often difficult and requires a formal risk–benefit analysis, even in the general population, in the absence of strong guideline recommendations. The purpose of this review is to detail the particularities of cardiac device use in CKD patients.

## 2. CIED in CKD Patients with HF

### 2.1. Cardiac Resynchronization Therapy

Current European Society of Cardiology (ESC) guidelines recommend the use of CRT for symptomatic patients with normal sinus node function, wide QRS complex with left bundle branch block morphology, and persistent severe left ventricular systolic dysfunction despite optimal pharmacological treatment [10]. The impact of renal function on the benefits of CRT has been largely debated, but we must bear in mind that all available data come from observational studies. The outcomes of CKD patients with CRT are mainly influenced by responder status (defined as an increase in left ventricular ejection fraction (LVEF) of at least 5% or a decrease in left ventricular end-systolic volume (LVESV) of more than 10%) or non-responder status. Whether it is the presence of CKD that directly influences patients’ response to CRT is still debated.

In a study that included 179 patients with a mean LVEF of 24%, patients with CKD stages 3–5 had a functional response to CRT similar to that of patients with normal renal function, which also translated into an improvement in HF symptomatology. Although overall mortality was higher in renal patients, those who qualified as CRT responders had extended long-term survival [11]. Moreover, in patients with severe HF, CRT was associated with a sustained improvement in renal function defined as an increase in eGFR by at least 10 mL/min/1.73 m^2^ in all CKD stages, leading to a significant reduction in mortality and in the necessity for cardiac mechanical support [12].

A larger study that included 588 patients with ischemic HF and CKD, concluded that CRT responders, especially with CKD stages 4 and 5, may experience improved eGFR and decreased short-term mortality rates. However, the analysis on mortality rates did not reach statistical significance in the long-term [13].

In another study (on 178 patients with a median LVEF of 25% and non-ischemic HF etiology), renal impairment did not significantly influence patients’ response to CRT, but mortality was 5.5 times higher in patients with CKD compared with patients with normal renal function. Again, improved renal function was only documented in CRT responders [14].

However, patients with advanced stages of CKD may have a less significant response to CRT [15]. In a retrospective study that included 798 patients from the entire CKD spectrum with LVEF below 35%, patients with more advanced CKD (stages 3–5) and CRT had lower survival rates and more hospitalizations for HF compared with those with CKD stages 1–2. There was a benefit in cardiac remodeling, with an improvement in the LVEF and LVESV across all stages, albeit less significant for those with moderate-to-severe renal dysfunction. The study showed a preservation of renal function after initiation of CRT in patients with eGFR < 60 mL/min/1.73 m^2^, with a small improvement in eGFR (0.8 mL/min from baseline) at 6 months in responders compared with non-responders [16]. There are also data reporting an all-cause mortality rate of 66% (HR: 1.66, 95% CI (1.37–2.02), *p* < 0.01, 0% I^2^) in CKD patients who received CRT, and it appeared that all-cause mortality rates decreased by 19% for every 10-unit increase in GFR (HR: 0.81, 95% CI (0.73–0.90), *p* < 0.01, 86% I^2^) [17]. In another observational study that followed 1000 patients with CRT for a mean period of 3.7 years, a higher mortality rate (hazard ratio (HR): 1.53; 95% confidence interval (CI): 1.26–1.86), higher cardiac mortality (HR: 1.55; 95% CI: 1.23–1.95), and increase in the number of admissions for HF was observed in CRT patients with moderate and severe CKD (eGFR < 60 mL/min/1.73 m^2^), but with a benefit in the survival rate and number of hospitalizations in patients with a CRT defibrillator (CRT-D) regardless of CKD severity (eGFR ≥ 60—HR: 0.65; 95% CI: 0.45–0.95; eGFR < 60—HR: 0.64; 95% CI: 0.48–0.85). Thus, one may conclude that CRT-D is a better choice than CRT-P in patients with renal disease [18]. In this study, the impact of CKD on the mortality rate was similar in patients receiving CRT and CRT-D [19,20].

In conclusion, available data suggest that, in CRT responders, there is a significant improvement in both the mortality rates and cardiac functional response. Moreover, CRT responders may experience an improvement in renal function. However, we must bear in mind that, in the advanced stages of CKD, these benefits are attenuated. On the one hand, left ventricular hypertrophy, myocardial fibrosis, activation of the renin–angiotensin–aldosterone (RAA) system, increased inflammatory status, and oxidative stress are several potential explanations for both the negative influence of CKD on the mortality rate and the decreased response to CRT in the advanced stages of CKD. On the other hand, an improvement in left ventricular systolic function in CRT responders enhances the renal blood flow, decreases venous congestion, and therefore leads to an increase in eGFR [12,13,17]. Table S1 (see Supplementary Files) summarizes the principal findings for CKD patients in studies on CRT.

### 2.2. CIED for Sudden Cardiac Death (SCD) Prevention in CKD Patients

ICD therapy has been traditionally used for both primary and secondary prevention of SCD in patients with ischemic HF who either had persistently reduced LVEF despite 3 months of optimal medical therapy or were survivors of ventricular arrhythmias causing hemodynamic instability. In both situations, ICD therapy reduces the risk of SCD and all-cause mortality [10]. In recent years, ICD therapy has also been approved for the prevention of SCD in patients with cardiomyopathies, in which case the decision is mainly driven by the extent of myocardial fibrosis [21]. However, the efficacy of ICD remains uncertain in patients with moderate to severe renal disease because of their exclusion from clinical trials and a paucity of evidence [22]. In addition, studies performed on hemodialysis (HD) patients, who were monitored with implantable loop recorders, concluded that the underlying causes of SCD were more frequent bradyarrhythmias [23,24]. These data are confirmed by a meta-analysis performed on 317 HD patients that concluded that the incidence of bradycardia/asystole was higher than that of ventricular arrhythmias [25].

The influence of ICD therapy on all-cause death was assessed in over 17,000 patients with CKD. ICD therapy led to a decrease in the overall mortality rate in patients who were at a high risk of SCD and received the device for primary prevention (adjusted HR = 0.65, 95% CI = 0.47–0.91, *p* < 0.05). However, in patients who had already had ICD therapy, the development of CKD increased the risk of all-cause mortality by 2.86 times [26]. Other studies suggest that the beneficial effect of survival of ICD therapy may be limited to early stages of CKD, with the loss of this effect in stages 4 and 5 [27]. The excess of mortality in ESRD patients with ICD can be partially explained by higher defibrillation thresholds, increased number of comorbidities, and a higher frequency of device-associated infections. Moreover, patients with CKD frequently present with left ventricular hypertrophy, fluid overload, electrolyte imbalance, and autonomic disorders, all leading to a higher rate of electric storms [27].

Evidence regarding the benefits of ICD therapy in CKD patients is still controversial. There are studies that suggest better survival rates with ICD therapy, irrespective of the type of indication [28], while others conclude a higher mortality rate and an elevated burden of antitachycardia pacing or shocks [29]. A major limitation comes from the fact that many studies combine patients with ICD in both primary and secondary prevention, with or without HF. Therefore, we believe it would be useful to have a separate view according to the indication.

#### 2.2.1. ICD for Primary Prevention of SCD

The main concern about the efficacy of ICD therapy in CKD patients comes from a meta-analysis that included three cornerstone trials on this matter—MADIT I, MADIT II, and SCD-HeFT—which concluded that the decline of glomerular filtration rate was associated with a lower benefit of survival. Moreover, ICD patients with an eGFR below 60 mL/min/1.73 m^2^ had lower survival rates when compared with those without or with mild renal disease and ICD and to those without ICD therapy and normal kidney function. These results can be explained partially by the presence of non-arrhythmic events causing death, higher defibrillation thresholds, or decreased ICD exposure of CKD patients secondary to a shorter life expectancy compared with the non-CKD population. However, there were no concerns regarding the safety of the device, as the renal function had no impact on ICD-related complications’ rates [30].

Hess et al. conducted a larger retrospective study on over 21,000 patients with CKD who underwent ICD implantation for primary prevention of SCD. The risk of mortality after the ICD procedure was significantly higher in patients with renal disease, and the difference between subgroups (by GFR) was evident 6 months after device placement: one-year unadjusted Kaplan–Meier death rates among patients without CKD, patients with a GFR 30–60, GFR < 30, and end-stage renal disease on dialysis categories were 4.4%, 9.1%, 20.2%, and 22.4%, respectively. Patients with CKD also experienced higher rates of periprocedural complications (i.e., hematomas). The burden of death was inversely proportional with renal function and doubled for patients with eGFR between 30 and 60 mL/min (hazard ratio (HR) 2.08, 95% confidence interval (CI) 1.99–2.18, *p* < 0.0001), while those with severe CKD (eGFR < 30 mL/min) had a 4.2 times higher risk of mortality (HR 4.20, 95% CI 3.92–4.50, *p* < 00001, respectively). ESRD patients had a 4.8 greater risk of death, displaying the worst prognosis (HR 4.80, 95% CI 4.46–5.17, *p* < 00001).

The authors suggested that CKD was not the only factor that increased the burden of mortality, as patients with renal impairment had significantly more comorbidities. While the severity of renal dysfunction was most strongly associated with the risk of death (end-stage renal disease on dialysis vs. GFR 30–60, HR 2.29, 95% CI 2.12–2.46, *p* < 00001), other factors were strongly connected with higher mortality, e.g., patients with impaired renal function were usually older, had a higher degree of systolic dysfunction (lower LVEF) or worse HF symptoms (NYHA class), and were more frequently diagnosed with diabetes mellitus, hyponatremia, or supraventricular tachycardia like atrial fibrillation/flutter [31].

In an observational study that included 5877 patients with CKD and LVEF < 40%, ICD therapy did not lower the rate of all-cause mortality, and was associated with an increased number of hospital admissions in relation to HF [32]. In another observational study that included 303 dialysis patients with LVEF < 35%, patients who received ICD therapy for primary prevention of SCD did not have a significant survival advantage (HR 0.87, 95% CI 0.66–1.13, log-rank *p* = 0.29) [33]. These results were also consistent with findings in dialysis patients with a LVEF > 35% [34].

#### 2.2.2. ICD for Secondary Prevention of SCD

Ventricular arrhythmias are the main cause of SCD in advanced CKD stages. Moreover, SCD has a higher prevalence in hemodialysis (HD) patients and is responsible for one-third of deaths in HD patients and two-thirds of deaths in patients with advanced CKD [35,36]. In a systematic review and meta-analysis that included almost 120,000 CKD patients, ICD therapy for secondary prevention of SCD was associated with a reduced risk of all-cause mortality (OR = 0.47; 95% CI, 0.40 to 0.55) [37]. The benefits of ICD therapy on survival were also evaluated in a study that included more than 41,000 dialysis patients who survived major cardiovascular events, such as sudden cardiac arrest (SCA) and ventricular tachycardia/fibrillation, and were followed for 8 years. In this cohort, 3.4% (1442) of patients received an ICD for the secondary prevention of SCD. Patients with ICD had a significantly lower short- and long-term mortality rate when compared with those without ICD. The increase in mortality rate was mainly driven by a higher comorbidity burden and a higher number of both infectious and embolic complications. The positive results for ICD therapy could be explained through an elevated arrhythmic load in ESRD patients and from the characteristics of those selected for receiving such a device, i.e., less comorbidities (low CCI), younger age, and fewer cardiac events [38].

The higher rates of infections, mainly endocarditis, and limitations with vascular access in dialysis patients makes the placement of a transvenous ICD difficult. A subcutaneous implantable cardioverter defibrillator (S-ICD) is safer in terms of infectious complications but is associated with an increased risk of death and higher rates of electrical shocks [39].

In conclusion, the benefits of ICD therapy in CKD patients are limited to the indication of secondary prevention of SCD. As previously anticipated, in CKD patients who qualify for ICD therapy and associated HF with a reduced ejection fraction, CRT-D may be superior to ICD. In a retrospective study that included over 1000 patients with CKD stages 3–5, including patients on dialysis, CRT-D was associated with a significantly lower mortality rate and a reduced number of hospitalizations for HF when compared with ICD therapy alone (HR 0.84; 95% CI: 0.78 to 0.91; *p* < 0.0001). The authors noted a tendency for a more rapid progression rate to end-stage renal disease in the ICD group, but without reaching statistical significance [40]. A subgroup analysis of the MADIT-CRT trial noted a better survival rate and fewer HF-related events in patients with moderate CKD who received CRT-D compared with those with ICD only. On the other hand, CRT-D did not improve the mortality rate in those with normal renal function but had a significant effect on reducing HF-related events [19].

Finally, the benefits of survival after CRT-D therapy over ICD only were confirmed in a meta-analysis performed on 13,095 patients, including dialysis patients. CRT-D had a favorable effect on cardiac remodeling and was associated with an increase in eGFR due to an increase in kidney perfusion and with reduced sympathetic overactivation, all of which led to a decreased burden of ventricular arrhythmic events [28]. These results were also supported by the aforementioned meta-analysis, performed on 120,000 CKD patients [37].

Therefore, CRT-D may be the preferred CIED in CKD patients who meet the established criteria. Table S2 (see Supplementary Files) summarizes the principal findings for CKD patients in studies on ICD and CRT-D.

### 2.3. Left Ventricular Assist Device (LVAD) and CKD

As previously mentioned, CKD is a well-known predictor for worse outcomes in patients with advanced HF. LVADs are recommended for bridge-to-transplantation, destination therapy, bridge-to-decision, or bridge-to-recovery in patients with end-stage HF, refractory to maximal tolerated medical therapy [41].

In a retrospective study that followed 213 patients in terms of post-LVAD outcomes according to preexisting moderate or severe renal dysfunction, mortality rates were higher in patients who experienced a decline in renal function before LVAD implantation, and the incidence of stroke or transient ischemic attack, as well as hospitalisations for HF, was also higher. Impaired renal function was also associated with right ventricular systolic dysfunction [42].

In a large cohort that included over 20,000 patients with LVAD, patients with CKD stages 4–5 had an increased risk of death when compared with early stages (OR: 1.33, CI: 1.16–1.50). In addition, the duration of hospital stays was longer, with greater financial costs and an increased necessity for transitional care services at discharge [43]. Moreover, patients with CKD had a significantly higher rate of renal replacement therapy post-LVAD therapy [44]. These results were also confirmed by a recently published systematic review and meta-analysis performed on over 26,000 patients with CKD [45].

Data regarding the long-term benefit of LVAD in patients with advanced CKD and HF are scarce. In a retrospective study that included 496 patients with CKD and 95 ESRD patients, dialysis patients had significantly higher mortality rates when compared with non-dialysis patients with CKD (3 49.5% vs. 30.2%, adjusted *p* = 0.009). However, dialysis did not increase the rate of complications like bleeding, pump thrombosis, ischemic or hemorrhagic stroke, sepsis, or infections [46].

These results are in line with another retrospective study conducted on over 400 patients, with LVAD used as a bridge to heart transplant. In patients with ESRD, the mortality rate post-LVAD was significantly higher and most of them died before receiving a heart transplant [47]. However, there was not strong enough evidence to support a causal effect between the increased post-LVAD mortality and ESRD. Complications such as infection, stroke, bleeding, older age, or comorbidities—more frequently seen in ESRD—could also increase the mortality and lead to a poor prognosis [48,49].

In an observational study performed on 131 patients (almost half of them with pre-existing CKD), LVAD implantation was associated with an improvement in kidney function at 1 month [50]. On the other hand, the coexistence of AKI at the moment of LVAD implantation was associated with significantly more in-hospital deaths, as well as more frequent LVAD-related complications, e.g., bleeding, sepsis, or discharge to a nursing facility [51]. The incidence of AKI post-LVAD implantation varied between 11% and 45%, and its occurrence was explained (among other factors) by prolonged hypovolemia, congestion, right ventricle failure, cardio-renal syndrome, vasoplegia, or hemolysis [52]. The occurrence of AKI and the need for RRT after LVAD was more prevalent in patients with preexisting CKD [44] and led to an increased mortality and a decreased renal function after 12 months [53].

In conclusion, in patients with advanced HF and CKD (particularly in advanced stages), shared decision making regarding LVAD treatment is advisable. Table S3 (see Supplementary files) summarizes the principal findings for CKD patients in studies on LVAD.

## 3. CIED for Bradyarrhythmia in CKD

The incidence of cardiac arrest due to bradyarrhythmias or asystole in dialysis patients varies between 10% and 30% and occurs more frequently in long interdialytic periods [4,54,55,56]. Moreover, bradycardia and asystole are the main mechanisms of SCD in end-stage renal disease patients [57]. In a prospective cohort study, Rautavaara et al. underlined the importance of continuous and/or in center electrocardiographic monitoring. In their analysis, bradyarrhythmias occurred especially during in-center HD and no bradycardia was found on ambulatory 12-lead electrocardiograms (ECGs). However, the rate of bradycardia findings was significantly higher in those with long PR or RR intervals on ambulatory 12-lead ECGs [56].

Data derived from observational studies concluded that the need for cardiac pacing is three times higher in dialysis patients. Moreover, hemodialysis is more frequently associated with the need for cardiac pacing when compared with peritoneal dialysis [58]. In a longitudinal retrospective study that included 260 ESRD patients on RRT, patients with cardiac pacemakers had a higher mortality burden, but cardio-stimulation was not independently associated with an increased risk of death, with factors such as infections or arrhythmic events being more common. Patients with dual-chamber pacemakers had a better survival rate. Moreover, device implantation before starting RRT was associated with better outcomes when compared with cardiac stimulation after the initiation of RRT, but these results were mainly driven by a higher comorbidity burden in dialysis patients [59].

## 4. CIED-Related Infections: Particularities in CKD Patients

CIED-related infections are associated with increased rates of mortality and morbidity, as well as with an increased financial burden on the healthcare system [60]. In recent years, there has been a two- to four-fold increase in the number of CIED infections. The risk of infections is higher in CRT recipients, especially in the first year after implantation or in patients with repeated procedures [60,61,62].

Many factors can contribute to and promote a device-related infection. These factors can either be patient-related (age, male sex, comorbidities such as diabetes, renal insufficiency or chronic obstructive pulmonary disease, medication like anticoagulation or immune-modulating therapy, medical history, or need for vascular access), linked to the procedure (improper antibiotic prophylaxis, reintervention or device replacement, duration, temporary pacing, operator experience, hematoma, or lead dislodgement), or device-associated (number of leads, dual- or single-chamber device, epicardial leads, or pocket). In a meta-analysis that included 26,172 patients, CKD and ESKD (including dialysis) were associated with a significant risk of CIED infections (OR = 3.02 (1.38–6.64) and OR = 8.73 (3.42–22.31), respectively). In addition, the risk increased with a number of comorbidities such as diabetes mellitus or congestive heart failure and with CKD-related conditions like central venous catheter presence or corticosteroid use [63].

In CIED-related infections, current guidelines recommend the complete extraction of the system with a complementary antibiotic therapy [60]. In a study that included 1420 patients (261 with CKD) who had undergone transvenous lead extraction, the presence of CKD and dialysis status did not affect the success of the procedure or complication rates. However, patients with CKD had a significantly worse survival at both 1- and 6-month follow-up [64]. Device extraction was superior to medical therapy in terms of survival among over 540,000 patients with CIED infection with ESRD [65].

Leadless CIEDs could be a useful alternative in CKD patients due to their difficult vascular access and higher liability for infections [66].

## 5. Conclusions

To conclude, the decision of implanting a cardiac device in a CKD patient should be personalized. In CKD patients and the general population alike, benefits and risks should be equally taken into consideration. In addition, in ESRD patients, the type of RRT may influence the risk of associated complications, especially infections. CRT is more effective in the early stages of CKD, in which case it is also associated with a higher rate of response. ICD has solid evidence for secondary prevention of SCD, while in primary prevention, the mortality rate is significantly increased when compared with the general population. LVADs are usually associated with an increased mortality rate in CKD patients, with limited evidence supporting an improvement in kidney function after their implant. Cardiac pacing indications are similar to those in the general population.

## Data Availability

No new data were created or analyzed in this study. Data sharing is not applicable to this article.

## Supplementary Materials

The following supporting information can be downloaded at: https://www.mdpi.com/article/10.3390/jcm13020516/s1, Table S1: Studies on CRT—main findings for CKD patients. Table S2: Studies on ICD—main findings for CKD patients. Table S3: Studies on LVAD—main findings for CKD patients.

## References

[1] Warrens H., Banerjee D., Herzog C.A. (2022). Cardiovascular Complications of Chronic Kidney Disease: An Introduction. Eur. Cardiol. Rev..

[2] House A.A., Wanner C., Sarnak M.J., Piña I.L., McIntyre C.W., Komenda P., Kasiske B.L., Deswal A., deFilippi C.R., Cleland J.G.F. (2019). Heart failure in chronic kidney disease: Conclusions from a Kidney Disease: Improving Global Outcomes (KDIGO) Controversies Conference. Kidney Int..

[3] Hakopian N.N., Gharibian D., Nashed M.M. (2019). Prognostic Impact of Chronic Kidney Disease in Patients with Heart Failure. Perm. J..

[4] Turakhia M.P., Blankestijn P.J., Carrero J.J., Clase C.M., Deo R., Herzog C.A., Kasner S.E., Passman R.S., Pecoits-Filho R., Reinecke H. (2018). Chronic kidney disease and arrhythmias: Conclusions from a Kidney Disease: Improving Global Outcomes (KDIGO) Controversies Conference. Eur. Heart J..

[5] Di Lullo L., House A., Gorini A., Santoboni A., Russo D., Ronco C. (2015). Chronic kidney disease and cardiovascular complications. Heart Fail. Rev..

[6] Naraen A., Duvva D., Rao A. (2023). Heart Failure and Cardiac Device Therapy: A Review of Current National Institute of Health and Care Excellence and European Society of Cardiology Guidelines. Arrhythmia Electrophysiol. Rev..

[7] Olsen T., Jørgensen O.D., Nielsen J.C., Thøgersen A.M., Philbert B.T., Frausing M.H.J.P., Sandgaard N.C.F., Johansen J.B. (2022). Risk factors for cardiac implantable electronic device infections: A nationwide Danish study. Eur. Heart J..

[8] Ferrari A.D.L., Oliveira E.B., Tagliari A.P., Kochi A.N., Beuren T.M.A., Cabral G.C., Ferreira F.V.C., Danzmann L.C. (2023). Cardiomyopathy Induced by Artificial Cardiac Pacing: To Whom, When, Why, and How? Insights on Heart Failure Development. Braz. J. Cardiovasc. Surg..

[9] Manzoor H., Bhatt H. (2023). Prerenal Kidney Failure. StatPearls.

[10] McDonagh T.A., Metra M., Adamo M., Gardner R.S., Baumbach A., Böhm M., Burri H., Butler J., Čelutkienė J., Chioncel O. (2021). 2021 ESC Guidelines for the diagnosis and treatment of acute and chronic heart failure: Developed by the Task Force for the diagnosis and treatment of acute and chronic heart failure of the European Society of Cardiology (ESC) With the special contribution of the Heart Failure Association (HFA) of the ESC. Eur. Heart J..

[11] Bogdan S., Klempfner R., Sabbag A., Luria D., Gurevitz O., Bar-Lev D., Lipchenca I., Nof E., Kuperstein R., Goldenberg I. (2014). Functional response to cardiac resynchronization therapy in patients with renal dysfunction and subsequent long-term mortality. J. Cardiovasc. Electrophysiol..

[12] Singal G., Upadhyay G.A., Borgquist R., Friedman D.J., Chatterjee N.A., Kandala J., Park M.Y., Orencole M., Dec G.W., Picard M.H. (2015). Renal Response in Patients with Chronic Kidney Disease Predicts Outcome Following Cardiac Resynchronization Therapy. Pacing Clin. Electrophysiol..

[13] Jeevanantham V., Turagam M., Shanberg D., Reddy M., Atoui M., Daubert J.P., Dawn B., Lakkireddy D. (2016). Cardiac Resynchronization Therapy prevents progression of renal failure in heart failure patients. Indian. Pacing Electrophysiol. J..

[14] Moreira R.I., Cunha P.S., Rio P., da Silva M.N., Branco L.M., Galrinho A., Feliciano J., Soares R., Ferreira R.C., Oliveira M.M. (2018). Response and outcomes of cardiac resynchronization therapy in patients with renal dysfunction. J. Interv. Card. Electrophysiol..

[15] Gronda E., Genovese S., Padeletti L., Cacciatore F., Vitale D.F., Bragato R., Innocenti L., Schiano C., Sommese L., De Pascale M.R. (2015). Renal function impairment predicts mortality in patients with chronic heart failure treated with resynchronization therapy. Cardiol. J..

[16] Ter Maaten J.M., Martens P., L’Hoyes W., Maass A.H., Damman K., Dupont M., Mullens W. (2019). Response to Cardiac Resynchronization Therapy Across Chronic Kidney Disease Stages. J. Card. Fail..

[17] Bazoukis G., Letsas K.P., Korantzopoulos P., Thomopoulos C., Vlachos K., Georgopoulos S., Karamichalakis N., Saplaouras A., Efremidis M., Sideris A. (2017). Impact of baseline renal function on all-cause mortality in patients who underwent cardiac resynchronization therapy: A systematic review and meta-analysis. J. Arrhythm..

[18] Leyva F., Zegard A., Taylor R., Foley P.W.X., Umar F., Patel K., Panting J., Ferro C.J., Chalil S., Marshall H. (2019). Renal function and the long-term clinical outcomes of cardiac resynchronization therapy with or without defibrillation. Pacing Clin. Electrophysiol..

[19] Daimee U.A., Moss A.J., Biton Y., Solomon S.D., Klein H.U., McNitt S., Polonsky B., Zareba W., Goldenberg I., Kutyifa V. (2015). Long-Term Outcomes with Cardiac Resynchronization Therapy in Patients with Mild Heart Failure with Moderate Renal Dysfunction. Circ. Heart Fail..

[20] Daly D.D., Maran A., Hyer J.M., Funke F., Waring A., Cuoco F.A., Sturdivant J.L., Leman R.B., Gold M.R. (2016). The Effect of Chronic Kidney Disease on Mortality with Cardiac Resynchronization Therapy. Pacing Clin. Electrophysiol..

[21] Zeppenfeld K., Tfelt-Hansen J., de Riva M., Winkel B.G., Behr E.R., Blom N.A., Charron P., Corrado D., Dagres N., de Chillou C. (2022). 2022 ESC Guidelines for the management of patients with ventricular arrhythmias and the prevention of sudden cardiac death: Developed by the task force for the management of patients with ventricular arrhythmias and the prevention of sudden cardiac death of the European Society of Cardiology (ESC) Endorsed by the Association for European Paediatric and Congenital Cardiology (AEPC). Eur. Heart J..

[22] Khan M.S., Ahmed A., Greene S.J., Fiuzat M., Kittleson M.M., Butler J., Bakris G.L., Fonarow G.C. (2023). Managing Heart Failure in Patients on Dialysis: State-of-the-Art Review. J. Card. Fail..

[23] Sacher F., Jesel L., Borni-Duval C., De Precigout V., Lavainne F., Bourdenx J.P., Haddj-Elmrabet A., Seigneuric B., Keller A., Ott J. (2018). Cardiac Rhythm Disturbances in Hemodialysis Patients: Early Detection Using an Implantable Loop Recorder and Correlation with Biological and Dialysis Parameters. JACC Clin. Electrophysiol..

[24] Roberts P.R., Zachariah D., Morgan J.M., Yue A.M., Greenwood E.F., Phillips P.C., Kalra P.A., Green D., Lewis R.J., Kalra P.R. (2017). Monitoring of arrhythmia and sudden death in a hemodialysis population: The CRASH-ILR Study. PLoS ONE.

[25] Roberts P.R., Stromberg K., Johnson L.C., Wiles B.M., Mavrakanas T.A., Charytan D.M. (2021). A Systematic Review of the Incidence of Arrhythmias in Hemodialysis Patients Undergoing Long-Term Monitoring with Implantable Loop Recorders. Kidney Int. Rep..

[26] Makki N., Swaminathan P.D., Hanmer J., Olshansky B. (2014). Do implantable cardioverter defibrillators improve survival in patients with chronic kidney disease at high risk of sudden cardiac death? A meta-analysis of observational studies. EP Eur..

[27] Fu L., Zhou Q., Zhu W., Lin H., Ding Y., Shen Y., Hu J., Hong K. (2017). Do Implantable Cardioverter Defibrillators Reduce Mortality in Patients with Chronic Kidney Disease at All Stages?. Int. Heart J..

[28] Shurrab M., Ko D.T., Zayed Y., Navaneethan S.D., Yadak N., Yaseen A., Kaoutskaia A., Qamhia W., Hamdan Z., Haj-Yahia S. (2018). Outcomes of ICDs and CRTs in patients with chronic kidney disease: A meta-analysis of 21,000 patients. J. Interv. Card. Electrophysiol..

[29] Kiage J.N., Latif Z., Craig M.A., Mansour N., Khouzam R.N. (2021). Implantable Cardioverter Defibrillators and Chronic Kidney Disease. Curr. Probl. Cardiol..

[30] Pun P.H., Al-Khatib S.M., Han J.Y., Edwards R., Bardy G.H., Bigger J.T., Buxton A.E., Moss A.J., Lee K.L., Steinman R. (2014). Implantable cardioverter-defibrillators for primary prevention of sudden cardiac death in CKD: A meta-analysis of patient-level data from 3 randomized trials. Am. J. Kidney Dis..

[31] Hess P.L., Hellkamp A.S., Peterson E.D., Sanders G.D., Al-Khalidi H.R., Curtis L.H., Hammill B.G., Pun P.H., Curtis J.P., Anstrom K.J. (2014). Survival after primary prevention implantable cardioverter-defibrillator placement among patients with chronic kidney disease. Circ. Arrhythm. Electrophysiol..

[32] Bansal N., Szpiro A., Reynolds K., Smith D.H., Magid D.J., Gurwitz J.H., Masoudi F., Greenlee R.T., Tabada G.H., Sung S.H. (2018). Long-term Outcomes Associated with Implantable Cardioverter Defibrillator in Adults with Chronic Kidney Disease. JAMA Intern. Med..

[33] Pun P.H., Hellkamp A.S., Sanders G.D., Middleton J.P., Hammill S.C., Al-Khalidi H.R., Curtis L.H., Fonarow G.C., Al-Khatib S.M. (2015). Primary prevention implantable cardioverter defibrillators in end-stage kidney disease patients on dialysis: A matched cohort study. Nephrol. Dial. Transplant..

[34] Jukema J.W., Timal R.J., Rotmans J.I., Hensen L.C.R., Buiten M.S., de Bie M.K., Putter H., Zwinderman A.H., van Erven L., Krol-van Straaten M.J. (2019). Prophylactic Use of Implantable Cardioverter-Defibrillators in the Prevention of Sudden Cardiac Death in Dialysis Patients. Circulation.

[35] Jankowski J., Floege J., Fliser D., Böhm M., Marx N. (2021). Cardiovascular Disease in Chronic Kidney Disease. Circulation.

[36] Wanner C., Herzog C.A., Turakhia M.P., Blankestijn P.J., Carrero J.-J., Clase C.M., Deo R., Kasner S.E., Passman R.S., Pecoits-Filho R. (2018). Chronic kidney disease and arrhythmias: Highlights from a Kidney Disease: Improving Global Outcomes (KDIGO) Controversies Conference. Kidney Int..

[37] Liu Y., Sun J.Y., Zhu Y.S., Li Z.M., Li K.L., Wang R.X. (2021). Association between CRT(D)/ICD and renal insufficiency: A systematic review and meta-analysis. Semin. Dial..

[38] Payne T., Waller J., Kheda M., Nahman N.S., Maalouf J., Gopal A., Hreibe H. (2020). Efficacy of Implantable Cardioverter-defibrillators for Secondary Prevention of Sudden Cardiac Death in Patients with End-stage Renal Disease. J. Innov. Card. Rhythm. Manag..

[39] El-Chami M.F., Burke M.C., Herre J.M., Shah M.H., Sadhu A., Niebauer M.J., Kutalek S.P., Carter N., Gold M.R. (2020). Outcomes of subcutaneous implantable cardioverter-defibrillator in dialysis patients: Results from the S-ICD post-approval study. Heart Rhythm..

[40] Friedman D.J., Singh J.P., Curtis J.P., Tang W.H.W., Bao H., Spatz E.S., Hernandez A.F., Patel U.D., Al-Khatib S.M. (2015). Comparative Effectiveness of CRT-D Versus Defibrillator Alone in HF Patients with Moderate-to-Severe Chronic Kidney Disease. J. Am. Coll. Cardiol..

[41] Frigerio M. (2021). Left Ventricular Assist Device: Indication, Timing, and Management. Heart Fail. Clin..

[42] Mohamedali B., Bhat G. (2017). The Influence of Pre-Left Ventricular Assist Device (LVAD) Implantation Glomerular Filtration Rate on Long-Term LVAD Outcomes. Heart Lung Circ..

[43] Doshi R., Taha M., Pisipati S., Patel K., Al-Khafaji J., Desai R., Shah J., Gullapalli N. (2020). Impact of chronic kidney disease on in-hospital outcomes following left ventricular assist device placement: A national perspective. Heart Lung.

[44] Ajmal M.S., Parikh U.M., Lamba H., Walther C. (2020). Chronic Kidney Disease and Acute Kidney Injury Outcomes Post Left Ventricular Assist Device Implant. Cureus.

[45] Ibrahim M., Saint Croix G.R., Lacy S., Chaparro S. (2021). Impact of Renal Dysfunction on Outcomes after Left Ventricular Assist Device: A Systematic Review. Int. J. Heart Fail..

[46] Dalia T., Chan W.C., Sauer A.J., Ranka S., Goyal A., Mastoris I., Pothuru S., Abicht T., Danter M., Vidic A. (2022). Outcomes in Patients with Chronic Kidney Disease and End-stage Renal Disease and Durable Left Ventricular Assist Device: Insights from the United States Renal Data System Database. J. Card. Fail..

[47] Bansal N., Hailpern S.M., Katz R., Hall Y.N., Kurella Tamura M., Kreuter W., O’Hare A.M. (2018). Outcomes Associated with Left Ventricular Assist Devices among Recipients with and without End-stage Renal Disease. JAMA Intern. Med..

[48] Lakhdar S., Nassar M., Buttar C., Guzman Perez L.M., Akbar S., Zafar A., Munira M. (2022). Outcomes with Left Ventricular Assist Device in End-Stage Renal Disease: A Systematic Review. Cureus.

[49] Shah Z., Vuddanda V., Masoomi R., Rali A., Gupta B., Haglund N., Abicht T., Sauer A. (2017). Recent Trends and Outcomes of LVAD Implantation in the ESRD Population: Analysis of 2010–2014 National Inpatient Sample Database. J. Card. Fail..

[50] Wettersten N., Estrella M., Brambatti M., Horiuchi Y., Adler E., Pretorius V., Murray P.T., Shlipak M., Ix J.H. (2021). Kidney Function Following Left Ventricular Assist Device Implantation: An Observational Cohort Study. Kidney Med..

[51] Silver S.A., Long J., Zheng Y., Goldstone A.B., Franz D., Chang T.I., Chertow G.M. (2020). Outcomes after left ventricular assist device implantation in patients with acute kidney injury. J. Thorac. Cardiovasc. Surg..

[52] Yalcin Y.C., Bunge J.J.H., Guven G., Muslem R., Szymanski M., den Uil C.A., Hesselink D.A., Topkara V.K., Manintveld O.C., Colombo P.C. (2019). Acute kidney injury following left ventricular assist device implantation: Contemporary insights and future perspectives. J. Heart Lung Transplant..

[53] Muslem R., Caliskan K., Akin S., Sharma K., Gilotra N.A., Constantinescu A.A., Houston B., Whitman G., Tedford R.J., Hesselink D.A. (2018). Acute kidney injury and 1-year mortality after left ventricular assist device implantation. J. Heart Lung Transplant..

[54] Wong M.C., Kalman J.M., Pedagogos E., Toussaint N., Vohra J.K., Sparks P.B., Sanders P., Kistler P.M., Halloran K., Lee G. (2015). Temporal distribution of arrhythmic events in chronic kidney disease: Highest incidence in the long interdialytic period. Heart Rhythm.

[55] Wan C., Herzog C.A., Zareba W., Szymkiewicz S.J. (2014). Sudden cardiac arrest in hemodialysis patients with wearable cardioverter defibrillator. Ann. Noninvasive Electrocardiol..

[56] Rautavaara J., Kerola T., Kaartinen K., Vilpakka M., Aitkoski A., Anttonen O., Ahvonen J., Koistinen J., Vääräniemi K., Miettinen M. (2022). Asystole episodes and bradycardia in patients with end-stage renal disease. Nephrol. Dial. Transplant..

[57] Wong M.C.G., Kalman J.M., Pedagogos E., Toussaint N., Vohra J.K., Sparks P.B., Sanders P., Kistler P.M., Halloran K., Lee G. (2015). Bradycardia and asystole is the predominant mechanism of sudden cardiac death in patients with chronic kidney disease. J. Am. Coll. Cardiol..

[58] Wang I.K., Lin K.H., Lin S.Y., Lin C.L., Chang C.T., Yen T.H., Sung F.C. (2016). Permanent cardiac pacing in patients with end-stage renal disease undergoing dialysis. Nephrol. Dial. Transplant..

[59] Vanerio G., García C., González C., Ferreiro A. (2014). Mortality in patients on renal replacement therapy and permanent cardiac pacemakers. Int. J. Nephrol..

[60] Blomström-Lundqvist C., Traykov V., Erba P.A., Burri H., Nielsen J.C., Bongiorni M.G., Poole J., Boriani G., Costa R., Deharo J.C. (2020). European Heart Rhythm Association (EHRA) international consensus document on how to prevent, diagnose, and treat cardiac implantable electronic device infections-endorsed by the Heart Rhythm Society (HRS), the Asia Pacific Heart Rhythm Society (APHRS), the Latin American Heart Rhythm Society (LAHRS), International Society for Cardiovascular Infectious Diseases (ISCVID) and the European Society of Clinical Microbiology and Infectious Diseases (ESCMID) in collaboration with the European Association for Cardio-Thoracic Surgery (EACTS). Europace.

[61] Joy P.S., Kumar G., Poole J.E., London B., Olshansky B. (2017). Cardiac implantable electronic device infections: Who is at greatest risk?. Heart Rhythm..

[62] Modi V., Shah K., Ferraro B., Gasimli-Gamache L., Nanda S., Stevens S., Shirani J. (2023). Cardiac implantable electronic device implantation and device-related infection. EP Eur..

[63] Polyzos K.A., Konstantelias A.A., Falagas M.E. (2015). Risk factors for cardiac implantable electronic device infection: A systematic review and meta-analysis. Europace.

[64] Barakat A.F., Wazni O.M., Tarakji K.G., Callahan T., Nimri N., Saliba W.I., Shah S., Abdur Rehman K., Rickard J., Brunner M.P. (2018). Transvenous Lead Extraction in Chronic Kidney Disease and Dialysis Patients with Infected Cardiac Devices. Circ. Arrhythm. Electrophysiol..

[65] Guha A., Maddox W.R., Colombo R., Nahman N.S., Kintziger K.W., Waller J.L., Diamond M., Murphy M., Kheda M., Litwin S.E. (2015). Cardiac implantable electronic device infection in patients with end-stage renal disease. Heart Rhythm..

[66] El-Chami M.F., Clementy N., Garweg C., Omar R., Duray G.Z., Gornick C.C., Leyva F., Sagi V., Piccini J.P., Soejima K. (2019). Leadless Pacemaker Implantation in Hemodialysis Patients: Experience with the Micra Transcatheter Pacemaker. JACC Clin. Electrophysiol..

