# Supplementary material for: Cardiac Device Therapy in Patients with Chronic Kidney Disease: An Update
Source: J Clin Med. 2024 Jan 17;13(2):516. doi: 10.3390/jcm13020516 (PMC10816721; doi:10.3390/jcm13020516)
Supplement: Supplementary file 1 [file jcm-13-00516-s001.zip › jcm-2766302-supplementary.pdf]

Study design	Population characteristics	Principal findings
J.M. Ter Maaten el. al, 2019<br>Retrospective study<br>N=798	Mean LVEF<35%<br>QRS>130 ms	- Low survival rates and more hospitalizations for HF in presence of CKD stage 3-5 compared to those with CKD 1-2<br>- Reverse cardiac remodeling across all stages, but with a lower response for those with moderate to severe renal dysfunction, especially in ESRD<br>- Coservation of renal function after initiation of CRT in patients with eGFR<60 ml/min/1,73 m^2 with a small improvement of eGFR at 6 months in responders compared with non-responders
Levya et al, 2019<br>Observational study<br>N=1046	LVEF<35%<br>Mean LVEF=25%<br>410-CRT-D<br>636-CRT-P	- High rate of mortality and number of admission for HF in CRT patients with moderate and severe CKD<br>- Benefit in survival and hospitalizations rates in patients with CRT-D in comparison to CRT-P
Bazokis et al, 2017<br>Systematic review<br>16 studies<br>N= 7080	Predominantly ischemic HF<br>NYHA II-IV<br>LVEF<30%	- Significant higher rate of all-cause mortality in those with renal impairment; increased the risk of mortality with 66%<br>- Improvement of glomerular filtration rate was associated with significant survival progress
Bogdan et al, 2014<br>Single centre restrospective study<br>N= 179	HF, mean LVEF=24%	- Low survival rate in patients with eGFR<60 ml/min/1,73 m^2<br>- Long-term survival benefit in CRT respinders (at least 5% incresing of LVEF)<br>- Renal impairment din not influence the clinical and functional response<br>- The functional progress was independently linked with rise in long-term rate of survival
Gronda et al, 2015<br>Single centre retrospecive study;<br>N= 375	mean LVEF=28%, mean QRS duration<br>0,154 s	- Higer mortality in patients with renal dysfunction (eGFR<60 ml/min/1,73 m^2)<br>- Increased eGFR at 6 months after CRT implant was significantly associated with better survival for each 10 mL/min/<br>- Baseline eGFR differed significantly between CRT responders (improved LVEF with >10%) and non-responders
Jeevaantham et al, 2016<br>Retrospective study<br>N=588	41% non-ischemic HF, LVEF<35%	- Benefit on mortality derived from prevention in renal failure progress<br>- Stabilization of renal function with no significant eGFR deterioration, but without significant long-term effect on mortality<br>- The most important benefit in eGFR improvement was showed in advanced CKD (stage 4 and 5) at 6 months of follow-up<br>- CRT responder (LVEF incresing with >5%) had a decreased mortality rate on short-term, without benefit on long-term
Singal et al, 2015<br>Single centre retrospective study<br>N=260	Mean LVEF=24%<br>Mean QRS width=0,16 s<br>LBBB=51%	- Improvement of renal function in all CKD stages in patients with severe HF who received CRT<br>- eGFR incresing was associated with a significant reduction of death, need for HT or necesity of(LVAD); the benefit was sustainable during 5 years follow-up<br>- Better results appeared in patients already on treatment with ACEi/ARB, in those with more advanced CKD and increased LVEF (but no significant LVEF improvement in CKD stage II-IV)
Moreira et al, 2018 Single centre study<br>N=178	34 % ischemic HF 92% NYHA II-IV median LVEF=25%	- Significantly lower survival in patients with eGFR below 60 ml/min at 12, 24 and 36 months; 5.5 times incresed risk of death<br>- CRT responders (>15% reduction in end-systolic diameter of LV) had a better survival than non-responders<br>- Renal response defined as >10 ml/min increase of eGFR was significant only in CRT responders<br>- The renal impairment did not significanlty influence the echocardiografic response to CRT
Daimee et al, 2015,<br>Retrospective analysis of MADTI-CRTD trial<br>N=1820	HF, LVEF ≤30%, wide QRS ≥0,13 s<br>1274 LBBB (32% CKD)<br>533 no-LBBB (35% CKD)	- Patients with CRT-D, LBBB and eGFR<60 ml/min, had a significant 2 fold higher risk of death than patients with mid-normal kidney function

Daly Jr. et al, 2016,<br>Retrospective analysis<br>N=432	HF (60,7% ischemic)<br>LVEF <35%<br>Mean QRS 0,155 s<br>57%-LBBB	- Patients without or with mild renal disease had long-term lower mortality rates in comparison to those with moderate/severe CKD, including dialysis

Table S1: Studies on CRT – main findings for CKD patients

CRT-cardiac resincronization therapy; **ICD**-implantable cardioverter defibrillator; **CRT-D**-cardiac resincronzation therapy with defibrillator, **CRT-P** cardiac resincronzation therapy with pacemaker; **CKD**-chronic kidney disease; **ESRD**-End Stage Renal Disease; **HD**-haemodialysis; **PD**-peritoneal dialysis; **LVEF**-left ventricle ejection fraction; **RRT**-renal replacement therapy; **eGFR**-estimated Glomerular Filtration Rate; **NYHA**-New York Heart Association, **LBBB**=Left Bundle Branch Block; ACEi/ARB- angiotensin-converting enzyme inhibitor/angiotensin receptor blocker; **N**=numebr of patients;**MI**-miocardial infarction **LVAD**-left ventricle assist divice; **AKI**-acute kidney injury;AKI-D=acute kidney injury requiring dilaysis; **TIA**= transient ischemic attack, **RVF**=right ventricle failure; **CHF**=congestive heart failure; **HT**-heart transplantation; **BTT**-bridge to transplant, **DT**-destination therapy, **KT**-kidney transplant, **PM**-pacemaker, **VF**=ventricular fibrillation, **VT**-ventricular tachycardia, **SCA**-sudden cardiac arrest

Device	Study design	Population characteristics	Principal findings
**ICD**<br><br>**(both primary and secondary prevention-no separation according to indication)**	Makki et all, 2014<br><br>Meta-analysis of 5 retrospective studies<br><br>N=17460	2854 –ICD recipients<br><br>14606 – control<br><br>LVEF<30%	- Significantly lower overall mortality in CKD patients with ICD
	Makki et all, 2014<br><br>Meta-analysis of 15 studies (13-retrospective, 2-prospective)<br><br>N=5233	Board spectrum of EF (25-58%)	- The risk of death was significantly increased by renal impairment (2.86 times higher in CKD group)
	Fu et al, 2017<br><br>Meta-analysis<br><br>11 studies<br><br>N=20196	LVEF<31%<br><br>5 studies included dialysis patients<br><br>ICD -4178<br><br>Non-ICD -16018	- Benefit in survival after ICD implantation in stage 3 patients, but did not indicate any advantage in CKD 4-5<br><br>- Patients with CKD were more exposed to electric storm and had a greater risk of SCD<br><br>- CKD stage 4 was an independent factor in predicting time to first appropriate internal electric shock
	Shurrab et al, 2018<br><br>Meta-analysis of 11 retrospective studies N=21136<br><br>7/11 studies-ICD vs non ICD (N=8041)	ICD-1419<br><br>Non-ICD-6622<br><br>4/7 studies – included dialysis patients<br><br>LVEF<30%	- ICD therapy was associated with better survival rates in patients with eGFR<60ml/min, inclusive dialysis
	Kiage et al<br><br>Systematic review<br><br>42 studies	LVEF<35%<br><br>CKD and ESRD	- CKD patients with ICD experienced more death events and CKD was associated also with elevated burden of antitachycardia pacing or shocks
**ICD**<br><br>**Primary prevention**	Pun et al, 2014<br><br>Systematic review and meta-analysis	Symptomatic HF with<br><br>LVEF<35%	- Decline of eGFR is associated with reduction of benefit on mortality for patients with ICD<br><br>- ICD patients with eGFR below 60 ml/min/1,73 m^2 had lower survival rates compared with those with no or mild renal disease and non-ICD

	Bansal et al, 2018 Noninterventional<br><br>cohort study<br><br>N=5,877	LVEF <40%<br><br>ICD-1556<br><br>No-ICD-4321	- No difference regarding all-cause mortality between ICD and no-ICD groups<br><br>- ICD was associated with elevated rate of hospitalisations for HF or for any-cause among CKD patients
	Pun et al, 2015 Propensity Match Analysis<br><br>N=172	LVEF<35%<br><br>Dialysis patients:<br><br>ICD=86<br><br>No-ICD=86	- ICD did not improve survival at 1 or 3 years in dialysis patients
	Joukema et al, 2019, RCT<br><br>N=188	patients on HD/PD with LVEF > 35%	- There was no associated benefit on survival rate with similar rate of ICD related complications
	Hess et al, 2014, retrospective study,<br><br>N=47,282	Prior MI and LVEF<30% or CHF and LVEF<35%	- Risk of mortality after ICD placement was significantly greater in patients with renal disease<br><br>- The burden of death was proportional with more affected GFR; the risk of death doubled for eGFR between 30-60 ml/min; those with severe CKD (eGFR < 30 ml/min) had an 4,2 times increased hazard for mortality in ESRD/dialysis patients with a 4.8 greater risk of death
**ICD Secondary Prevention**	Liu et al, 2021 Systematic review<br><br>and meta-analysis<br><br>26 studies overall<br><br>N=199,263 patients	LVEF<35%<br><br>5/26 studies – ICD for<br><br>secondary prevention	- Reduced risk of all-cause mortality in patients with renal insufficiency with ICD for secondary prevention of SCD than in the non-ICD group<br><br>- CKD significantly increases the mortality of any cause
	Payne et al<br><br>Retrospective analysis<br><br>N=43,519	HD or PD patients who survived<br><br>VF,VT, SCA<br><br>3,4% (1,442) received ICD	- Rate of survival was considerable higher in the ICD group versus no-ICD (53% vs 27%); 46% decreased hazard of death and significant prolonged life expectancy (2,19 years versus 0,58 years)<br><br>- Principal risk factors for increased probability of mortality were higher burden of comorbidities (higher Charlson Comorbidity Index-CCI), septic events, pulmonary embolism, haemodialysis as initiation method of dialysis, predominantly for those on venous catheter or arteriovenous graft than on arteriovenous fistula, smoker status, alcohol use or hepatitis (B or C)
**CRT-D**<br><br>**Vs**<br><br>**ICD only**	Friedman et al, 2015, Retrospective study,<br><br>N=10946<br><br>CRTD-9525, ICD-1421	LVEF <35%<br><br>QRS > 0,12 s	- CRT-D signficatly decreased mortality and and hospitalisations for HF compared to ICD alone, across CKD 3-5D stages<br><br>- No difference in complications for CRT-D therapy comparative to ICD<br><br>- No significant difference in rate of proggresion to end-stage renal disease
	Liu et al, 2021<br><br>Systematic review and	LVEF<35%<br><br>6/26 studies – CRT-D VS ICD	- CRT-D therapy improves survival in comparison to ICD alone in patients with chronic renal disease<br><br>- Lower survival rate in dialysis patients compared to non-dialysis for both CRT-D or ICD patients

	Meta-analysis<br><br>26 studies overall<br><br>N=199263 patients		
	Shurrab et al, 2018<br><br>Meta-analysis of 11 retrospective studies N=21136<br><br>4/11 studies-CRT-D vs ICD only (N=13095)	CRT-D-10838<br><br>ICD only -2257<br><br>3/4 studies – included dialysis patients<br><br>LVEF<27%	- Significant benefit on survival from CRT-D therapy ofer ICD only in CKD patients, including dialysis
	Daimee et al, 2015,<br><br>Retrospective analysis of MADTI-CRTD trial<br><br>N=1820	HF, LVEF ≤30%, wide QRS ≥0,13 s<br><br>1274 LBBB (32% CKD)<br><br>533 no-LBBB (35% CKD)	- LBBB patients and moderate kidney dysfunction who received CRT-D had a significant better survival and less HF related events comparing to those with ICD only<br><br>- CRT-D din not improve rate of mortality in those with normal renal function, but had an significant effect on reducing events caused by HF<br><br>- Non-LBBB patients had not survival benefit from CRT-D over ICD only, no matter of eGFR

Table S2: Studies on ICD – main findings for CKD patients

CRT-cardiac resincronization therapy; **ICD**-implantable cardioverter defibrillator; **CRT-D**-cardiac resincronzation therapy with defibrillator, **CRT-P** cardiac resincronzation therapy with pacemaker; **CKD**-chronic kidney disease; **ESRD**-End Stage Renal Disease; **HD**-haemodialysis; **PD**-peritoneal dialysis; **LVEF**-left ventricle ejection fraction; **RRT**-renal replacement therapy; **eGFR**-estimated Glomerular Filtration Rate; **NYHA**-New York Heart Association, **LBBB**=Left Bundle Branch Block; ACEi/ARB- angiotensin-converting enzyme inhibitor/angiotensin receptor blocker; **N**=numebr of patients;**MI**-miocardial infarction **LVAD**-left ventricle assist divice; **AKI**-acute kidney injury;AKI-D=acute kidney injury requiring dilaysis; **TIA**= transient ischemic attack, **RVF**=right ventricle failure; **CHF**=congestive heart failure; **HT**-heart transplantation; **BTT**-bridge to transplant, **DT**-destination therapy, **KT**-kidney transplant, **PM**-pacemaker, **VF**=ventricular fibrillation, **VT**-ventricular tachycardia, **SCA**-sudden cardiac arrest

Devices	Study design	Population characteristics	Principal findings
**LVAD**<br><br>**And CKD/ESRD**	Mohamedali et al, 2017, Single centre retrospective study<br><br>N=213	Advaced HF patients<br><br>LVAD indicated as BTT or DT	- Significantly higher mortality in patients with eGFR<60m/min before LVAD implant compared to those with normal renal function<br><br>- Higher incidence of stroke or TIA, hospitalisations for HF and RVF in patients with eGFR<60 ml/min pre-LVAD<br><br>- No-improvement in renal dysfunction increased mortality<br><br>- Those with improvement in eGFR (post-LVAD) or absence of depressed kidney function pre and post-LVAD had a low incidence of RVF and HF admission<br><br>- Incidence of stroke was higher in those with baseline eGFR bellow 60 ml/min, irrespective of renal function evolution
	Doshi et al., 2020,<br><br>Retrospective observatonal study<br><br>N=20656	Stage D HF<br><br>All CKD stages, including HD/PD	- Increased risk of death in stages IV-V or ESRD patients compared to early stages of CKD (I-III)<br><br>- CKD patients had longer hospitalization periods, greater financial costs and increased necessity for transitional care services at discharge
	Ajmal et al.,2020<br><br>Retrospective study<br><br>N=137	LVAD for Class III or IV NYHA HF (ischemic and non-ischemic)	- Higher morality in patients requiring LVAD with CKD at baseline
	Ibrahim et al, 2021,<br><br>Systematic review,<br><br>7 studies<br><br>N=26652	4630 patients with renal dysfunction (eGFR<60ml/min)<br><br>LVAD for<br><br>advanced HF	- High risk of death in patients with chronic renal disease
	Bansal et. al,2018,<br><br>Retrospective study<br><br>N=416<br><br>ESRD=155;nonERD=261	LVAD used fot BTT or DT<br><br>ESRD:HD, PD and KT	- Patients in CKD final stage had significantly increased mortality (81,9% vs 36,4%) in comparison with non-ESRD group<br><br>- Most ESRD patients died in the first 3 weeks<br><br>- Most ESRD died without having received a heart transplantation<br><br>- In non-ESRD group, only 25% of patients received HT
	Dalia et al, 2022<br><br>Retrospective sutdy<br><br>N=591	496 patients with CKD, 95 patients with dialysis/ESRD underwent LVAD	- Dialysis (ESRD) patients had a significantly higher mortality rates when compared to patients with CKD (without need for dialysis)

	Lakhdar et al, 2022,<br><br>Systematic Review<br><br>13 articles	ESRD patients with LVAD as BBT or DT	- Increased LVAD use in ESRD patients (from 3,3 % in 2010 to 5,2 % in 2014)<br><br>- Decrease in mortality incidence (from 68% in 2010 to 54% in 2014)<br><br>- Generally higher rate of death in ESRD patients compared with no-ESRD<br><br>- No clear or direct association between mortality and ESRD; LVAD complications, older age and comorbidities could be also factors leading to increased mortality and poor prognosis
**LVAD and Acute Kidney Injury**	Wettersten et al, 2021<br><br>Observational study<br><br>N=131	47% of patients – pre-exsiting CKD	- LVAD implantation was associated with an improvement of kidney function at 1 month
	Silver et al, 2020,<br><br>Retrospective study<br><br>N=8362 patients	LVAD patients noHD AKI -3760<br><br>AKI-D - 436	- LVAD implantation is associated with significant more in-hospital deaths<br><br>- LVAD related complications: bleeding, sepsis or discharge to a nursing facility
	Muslem et al, 2018,<br><br>Retrospective multicentre study<br><br>N=241	LVAD implantation<br><br>169 (70%) developed AKI	- Development of acute kidney injury after LVAD implantation is also associated with higher mortality and decreased renal function at 12 months distance

Table S3: Studies on LVAD – main findings for CKD patients

CRT-cardiac resincronization therapy; **ICD**-implantable cardioverter defibrillator; **CRT-D**-cardiac resincronzation therapy with defibrillator, **CRT-P** cardiac resincronzation therapy with pacemaker; **CKD**-chronic kidney disease; **ESRD**-End Stage Renal Disease; **HD**-haemodialysis; **PD**-peritoneal dialysis; **LVEF**-left ventricle ejection fraction; **RRT**-renal replacement therapy; **eGFR**-estimated Glomerular Filtration Rate; **NYHA**-New York Heart Association, **LBBB**=Left Bundle Branch Block; ACEi/ARB- angiotensin-converting enzyme inhibitor/angiotensin receptor blocker; **N**=numebr of patients;**MI**-miocardial infarction **LVAD**-left ventricle assist divice; **AKI**-acute kidney injury;AKI-D=acute kidney injury requiring dilaysis; **TIA**= transient ischemic attack, **RVF**=right ventricle failure; **CHF**=congestive heart failure; **HT**-heart transplantation; **BTT**-bridge to transplant, **DT**-destination therapy, **KT**-kidney transplant, **PM**-pacemaker, **VF**=ventricular fibrillation, **VT**-ventricular tachycardia, **SCA**-sudden cardiac arrest
